# Supplementary material for: Caught between relief and unease: How university students’ well-being relates to their learning environment during the COVID-19 pandemic in the Netherlands
Source: PLoS One. 2023 Nov 2;18(11):e0292995. doi: 10.1371/journal.pone.0292995 (PMC10621861; doi:10.1371/journal.pone.0292995)
Supplement: S1 File — Including the interview guides and the diary template, (Table S1-S6). (PDF) [file pone.0292995.s001.pdf]

Supporting Information I - Materials

Interview Guides

The Tables S1 – S4 depict the interview guides for the four times of measurement, for the main interview after the self-presentation and explanation about the scope of the project, for both the student and faculty participants. Table S5 displays the interview guide for the focus group.

Diary Template

Table S6 illustrates the template for the diary that the students were asked to keep. There was an introductory text attached to the document:

> Thanks for participating in this diary study, accompanying the interview study on the effects of the Corona Crisis on student well-being. You agreed to provide us with more detailed insights into how the Corona Crisis affects your well-being, also in between the scheduled interviews. Then, one week before the next interview will be scheduled, please send this document back to the researcher. The content will be part of the following interview. Using this procedure, we hope to be able to find more profound data which helps us to understand student well-being in times of crisis.
>
> In specific:
>
> We would like you to fill out this short diary once a week. It is the easiest if you pick one situation which was challenging for you in this week. Please tell us, what exactly how the situation was (1), what made it so challenging (2), how you dealt with it (3), and finally, how it turned out in the end (4).
>
> If you want to, you can also include a photo which stands symbolically for something you experienced. Of course, you can always add thoughts of yours which you think might fit the topic, just like experiences and feelings that seem essential for you.
>
> In the following, you will have one page for every week. Like that, we hope that it will be easier for you to keep track. If you do not manage to fill in the diary every week, this will not pose any

problem. You can still withdraw from the study at any time without giving reasons. That also applies for parts of the interview study, such as the diary study. If you want to continue with the interview study, but not with writing a short diary, this will not result in any consequences. Just let the researchers know and we will proceed with the interview study as planned.

Apart from that, all regulations regarding privacy and anonymity you already consented to, counts for this document as well. If you have any further questions regarding this, do not hesitate to contact the researchers.

Thank you for your participation!

Tables

Table S1. ***Interview guide of the first time of measurement, for both students and faculty members.***

Topic	Students	Faculty
Student Well-being	I am really interested in how you experience your life at university, so could you please tell me about your studies and how you experienced them so far?	UT: I am really interested in what you think about how your students experience their life at the university, so could you please tell me about your experiences with student well-being so far?<br>Sta: I am really interested in what you think how students experience their life at the university, so could you please tell me about your experiences with student well-being so far?<br>Do you have any positive examples when it comes to student well-being?
	Can you tell me what well-being at the university means to you?	Can you tell me what student well-being at the university means to you?
	If I asked your professors what, in their opinion, contributes to student well-being, what do you think they would say?	If I asked students what, in their opinion, contributes to their well-being, what do you think they would say?
	How do you think other people influence your well-being positively?	How do you think other people influence student well-being positively?
Learning Environment	If a friend of yours was struggling with his/her studies, what would you recommend him/her to do?	UT: If a student of yours was struggling with his studies, what would you recommend him/her to do?<br>Sta: If a student was struggling and asked you for advice, what would you recommend him/her to do?
	Who is in your opinion responsible for student well-being?	Who is in your opinion responsible for student well-being?
	If you could distribute percentages, how would that look like?	If you could distribute percentages, how would that look like?
	Would you describe for me a situation in which you have felt supported by your department or the university in general?	How is student well-being incorporated within the daily life at the university?
	What does your department and the university in general do to enhance student well-being, that you are aware of?	What does your department and the university in general do to enhance student well-being, that you are aware of?

	What of those offers you just mentioned have you made use of?<br>What would you like your teachers, your department, or the university to do beyond that to prevent students from facing distress?<br>What do you think they need for that?	What experiences do you have with any of these offers you just mentioned?<br>What would you want to do beyond what is already done to prevent students from facing distress?<br>What would you need for doing that?<br>What would you like your department and the university to do?
	If you went to sleep tonight and tomorrow morning the university would miraculously have turned into an institution where everyone is satisfied with their studies. What would have changed?	If you went to sleep tonight and tomorrow morning the university would miraculously have turned into an institution where everyone is satisfied with their studies. What would have changed?<br>Not only students are important but also university staff. How do you personally perceive your well-being and staff well-being in general at the university?<br>How do your students influence your well-being?<br>What can you tell me about what the university does in order to enhance your well-being at the workplace?
Resilience	Now, this was more general towards student well-being. For the next part of the interview I am curious to hear about yourself and how you manage your well-being. So first, please tell me what you do next to your studies?	Now, this was more general towards student well-being. For the next part of the interview I am curious to hear about what you think about specific strategies that help managing someone's well-being. So first, please tell me what you do when you are facing distress?<br>And how does this differ from what students should do when they are feeling stressed out according to you?
	What do you do when you are feeling stressed out?<br>Can you tell me about a situation where it went really well when being stressed out?	
	I am really interested in how you manage to stay well during your studies. You already mentioned some things that make you feel good. Do you think we could try to categorize them according to XY?	I am really interested in how students could manage to stay well during their studies. You already mentioned some things that could make them feel good. Do you think we could try to categorize them according to XY?

	What kind of strategies do you use the most?<br>Can you tell me your top three strategies regarding your studies?	What kind of factors do you see are used most?<br>Can you tell me your top three factors?
Ending	So, the main points of what I took with me from your interview are XY. Is that right or did I misunderstood something?<br>Do you have anything more to say?<br>How did you experiences the interview?	So, the main points of what I took with me from your interview are XY. Is that right or did I misunderstood something?<br>Do you have anything more to say?<br>How did you experiences the interview?

Note. UT = university teacher, Sta = support staff

Table S2. ***Interview guide of the second time of measurement, for both students and faculty members.***

Topic	Students	Faculty
Student Well-being	How does a normal day of you look like at the moment?<br>How are you experiencing studying now compared to before the Corona Crisis?<br>How has student well-being changed due to the Corona Crisis?<br>Is there anything positive you learned about yourself now in this crisis?<br>When everything goes back to a normal state, which aspect of your life you developed for yourself for coping with the Corona Crisis do you want to keep?<br>If I asked your teacher how the Corona Crisis changed studying, what do you think they would say?	How does a normal day of a student look like at the moment?<br>How do you think studying has changed compared to before the Corona Crisis?<br>How has student well-being changed due to the Corona Crisis?<br>What could be something positive that students could have learned about themselves now in this crisis?<br>If I asked your students how the Corona Crisis changed studying, what do you think they would say?<br>How did your working life change? How do you handle home office, if I may ask?
Learning Environment	What do you think about how the university handles the situation?<br>What do you know about what your university does for your well-being in the current situation?<br>What would you need from the university in the following months?	What do you think about how the university handles the situation?<br>What do you know about what your university does for student well-being in the current situation?<br>What should be done beyond that?<br>How do you think education will have changed on the long run after the Corona Crisis?

Resilience	What helps you at the moment to maintain a positive state of well-being?<br><br>How do you handle negative experiences, thoughts, and emotions?<br><br>When you look at the resilience factors you stated the last time, which ones do you perceive as more important at the moment? Why?<br>Explain the effects of ...<br>1) Autonomy (example: detaching, spare time activities, mindfulness, keeping boundaries)<br>2) Competence (motivation regulation such as prioritizing and to-do-lists, persistence, doing things that make you feel competent)<br>3) Relatedness (social as well as academic support)<br>How do you think these factors have changed now due to the Corona Crisis?	What could help students at the moment to maintain a positive state of well-being?<br>How do you think they could handle negative experiences, thoughts, and emotions?<br>When you look at the resilience factors you stated the last time, which ones do you perceive as more important at the moment? Why?<br>Explain the effects of ...<br>1) Autonomy (example: detaching, spare time activities, mindfulness, keeping boundaries)<br>2) Competence (motivation regulation such as prioritizing and to-do-lists, persistence, doing things that make you feel competent)<br>3) Relatedness (social as well as academic support)<br>How do you think these factors have changed now due to the Corona Crisis?
Ending	How do you think the situation will have changed until July, when we will speak next?<br>Do you still have any questions?<br>Do you have anything more to say?<br>Did you wanted to answer anything I did not ask?	How do you think the situation will have changed until July, when we will speak next?<br>Do you still have any questions?<br>Do you have anything more to say?<br>Did you wanted to answer anything I did not ask?

Table S3. ***Interview guide of the third time of measurement, for both students and faculty members.***

Topic	Students	Faculty
Student Well-being	Individual questions concerning what they said last time about their well-being<br><br>Last time I asked you about what a normal day of you looks like. Did anything change since then in your daily routine?	Individual questions concerning what they said last time about student well-being<br><br>How do you think the daily life of a student has changed since the last time we spoke?

	How have you adapted to the new way of studying?	How do you think that students adapted to the new way of studying/counselling?
	How has student well-being changed since we last spoke? Has anything happened concerning student well-being that changed anything in your perception?	How has student well-being changed since we last spoke?
	If I asked your teacher how the Corona Crisis changed studying, what do you think they would say?	If I asked your students how the Corona Crisis changed studying, what do you think they would say?
	Is there anything positive you learned about yourself now in this crisis?	How have you personally adapted to the situation since the last time we spoke?
	When everything goes back to a normal state, which aspect of your life you developed for yourself for coping with the Corona Crisis do you want to keep?	How would you rank your well-being from a scale from 1-10 compared to t1 and t2?
		How do you feel about the prospective of teaching in the next academic year?
Learning Environment	How do you think the learning environment and studying experience changed throughout the last months?	How do you think the learning environment and studying experience changed for the students throughout the last months?
	What does the contact with your professor look like at the moment?	
	What do you need from the university right now for a better well-being?	What do you think students need from the university right now for a better well-being?
	How do you think education has already changed on the long run due to the experiences of these last months?	How do you think education already has changed on the long run through these last months?
Resilience	Individual questions concerning what they said about their resilience factors	Individual questions concerning what they said last time about resilience factors
	How do you implement your coping strategies nowadays?	How were your experiences with student well-being these last months?
	How has your social network changed these last months?	How do you think the students' social network might have changed?
Ending	How do you think the situation will have changed until July, when we will speak next?	How do you think the situation will have changed until July, when we will speak next?
	Do you still have any questions?	Do you still have any questions?

	Do you have anything more to say?<br>Did you wanted to answer anything I did not ask?	Do you have anything more to say?<br>Did you wanted to answer anything I did not ask?

Table S4. ***Interview guide of the fourth time of measurement, for both students and faculty members.***

Topic	Students	Faculty
Student Well-being	Individual questions concerning what they said last time about their well-being<br>How did you experience the beginning of the new term?<br>What were your experiences with your as well as your fellow students' well-being since the last time we spoke?<br>Which positive aspects that you – or other fellow students – have learned and experienced during the last couple of months are still noticeable?<br>How do you think experiencing a time like this might help you face adversities in the future?<br>How has student well-being changed compared to t1?	Individual questions concerning what they said last time about student well-being<br>How do you believe the students experienced the beginning of this term?<br>What were your experiences with student well-being since the last time we spoke?<br>Which positive aspects that students have learned and experienced during the last couple of months are still noticeable?<br>How do you think experiencing a time like this might help students face adversities in their future?<br>How are you personally experiencing working at the university compared to t1?<br>How has teaching/counselling changed compared to t1 due to the last couple of months?
Learning Environment	Now that you attend another university, do you see any differences within the learning environment compared to the RUG?<br>What does the contact with your new professors look like (compared to t1/compared to RUG)?<br>How connected do you feel to your new fellow students?<br>What did the university do to ensure connectedness to your fellow students/professors/faculty/university?	How does studying and the learning environment look like at the moment compared to t1?<br>How is your contact with the students compared to t1 and how did this change?<br>What do you think contributed to this?<br>What do you think students need from the university right now for a better well-being?

	What could the university do to support students for the upcoming period?	What could the university, or you as part of the university, do to support students for the upcoming period?
	Which positive aspects that the university developed during the last couple of months are still noticeable?	Which positive aspects that the university developed during the last couple of months are still noticeable?
Resilience	How do you implement your coping strategies nowadays?	
	Which of these aspects do you think students learned during this pandemic to better cope with future stressors? (One last look at the resilience factors to check for any changes)	Which of these aspects do you think students learned during this pandemic to better cope with future stressors? (One last look at the resilience factors to check for any changes)
Ending	Do you still have any questions?<br>Do you have anything more to say?<br>Did you wanted to answer anything I did not ask?	Do you still have any questions?<br>Do you have anything more to say?<br>Did you wanted to answer anything I did not ask?

Table S5. ***Interview guide of the focus group.***

Topic	Students	Faculty
Results third Research Question	Research question: The potential of the learning environment in impacting student well-being;<br>Explain the headlines really short (without adaptation) -> Jam board 5 minutes<br>**Where do you see the most potential for practical implications based on these results?**	
Link Student-Teacher	As you can see, the relationship between students and teachers are of significant importance. However, that has greatly been impacted due to the COVID crisis resulting in a distance between both parties, without a proper established or maintained relationship and without knowledge of the other's situation. That hits the basic psychological need for relatedness, which appeared to be highly compromised in these times. **With this in mind, next to my first paper stating that the well-being of both parties impact each other reciprocally, how do you perceive the significance of the teacher-student relationship within the setup of higher education studies?**	
Potentially: New normal	**Everybody is talking about a 'new normal' – how would you redesign education to become this new normal?**	
Ending	Do you still have any questions?<br>Did you wanted to answer anything I did not ask?	

Table S6. ***Template for the diary the students were asked to kept.***

Calendar Week	Dates of the week
We would like you to fill out this short diary once a week. It is the easiest if you pick one situation which was challenging for you in this week. Please tell us, what exactly how the situation was (1), what made it so challenging (2), how you dealt with it (3), and finally, how it turned out in the end (4).<br>(1)<br><br>(2)<br><br>(3)<br><br>(4)	
If you want to, you can also include a photo which stands symbolically for something you experienced. Of course, you can always add thoughts of yours which you think might fit the topic, just like experiences and feelings that seem essential for you.	
