# Supplementary material for: Caught between relief and unease: How university students’ well-being relates to their learning environment during the COVID-19 pandemic in the Netherlands
Source: PLoS One. 2023 Nov 2;18(11):e0292995. doi: 10.1371/journal.pone.0292995 (PMC10621861; doi:10.1371/journal.pone.0292995)
Supplement: S2 File — Including the categorisations identified during the interviews and discussed during the focus group (Table Figure S1 & S2). (PDF) [file pone.0292995.s002.pdf]

Supporting Information II - Results

Supporting Information Regarding the Results

Categorisations Identified During the Interviews

During the first interview, all participants created categorisation of resilience factors based on the preceding interview. At t2, they rated how these resilience factors have become more or less important or changed in nature. At t4, participants were asked to determine which resilience factors bared the potential for resilience growth. Figure S1 depicts all six categorisations.

Results From the Focus Group

Figure S2 illustrates the participants initial reactions to the preliminary findings regarding the third research question focusing on the learning environment.

Figures

Figure S1. Categorisations of all both students (a and b), both teachers (c and d), the study advisor (e), and the student psychologist (f).

(a)

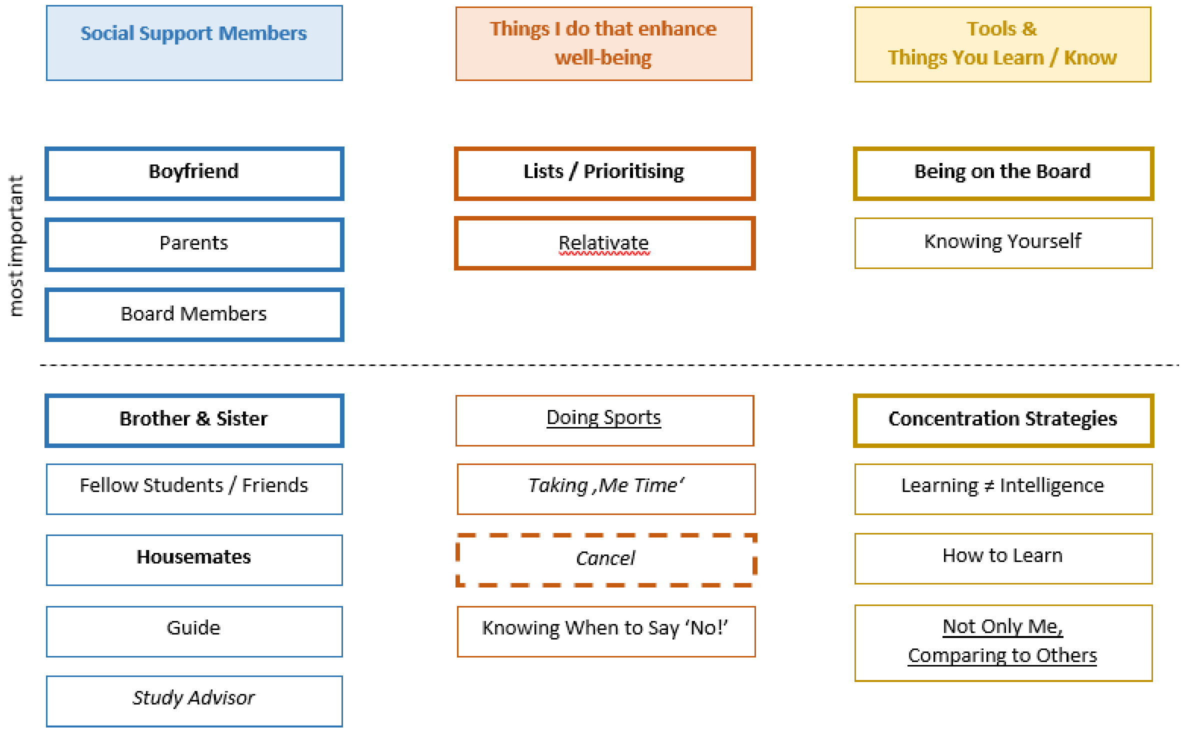

(b)

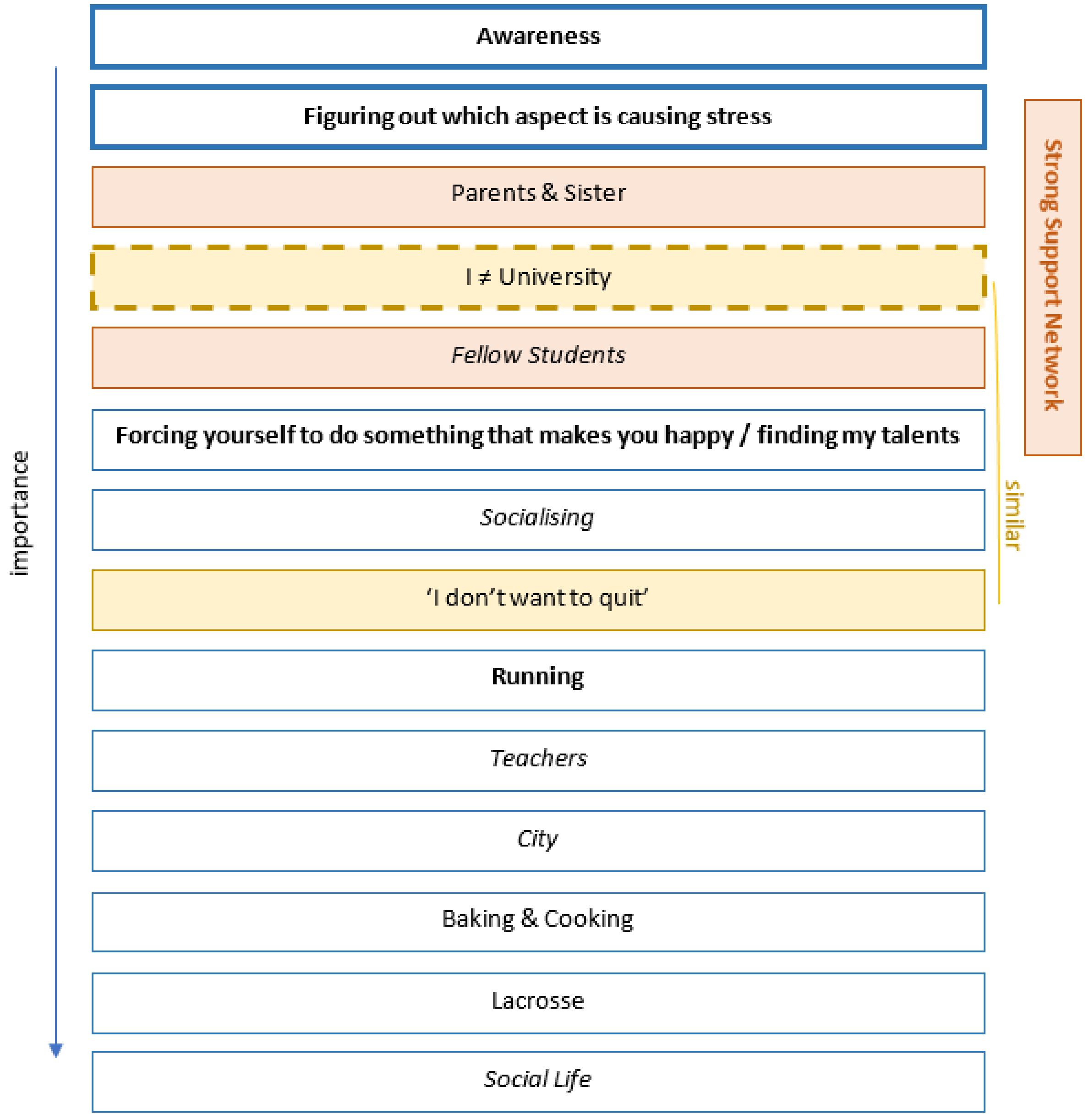
Awareness
Figuring out which aspect is causing stress
Parents & Sister
I ≠ University
Fellow Students
Forcing yourself to do something that makes you happy / finding my talents
Socialising
'I don't want to quit'
Running
Teachers
City
Baking & Cooking
Lacrosse
Social Life
importance
similar
Strong Support Network

(c)

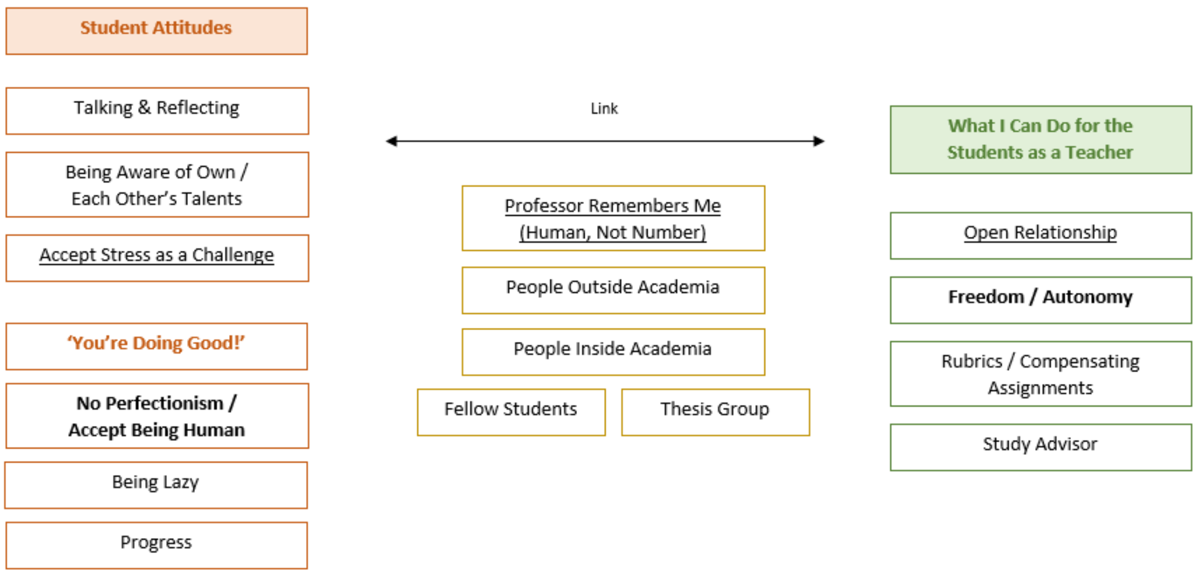
Student Attitudes
Talking & Reflecting
Being Aware of Own / Each Other's Talents
Accept Stress as a Challenge
'You're Doing Good!'
No Perfectionism / Accept Being Human
Being Lazy
Progress
Link
Professor Remembers Me (Human, Not Number)
People Outside Academia
People Inside Academia
Fellow Students
Thesis Group
What I Can Do for the Students as a Teacher
Open Relationship
Freedom / Autonomy
Rubrics / Compensating Assignments
Study Advisor

(d)

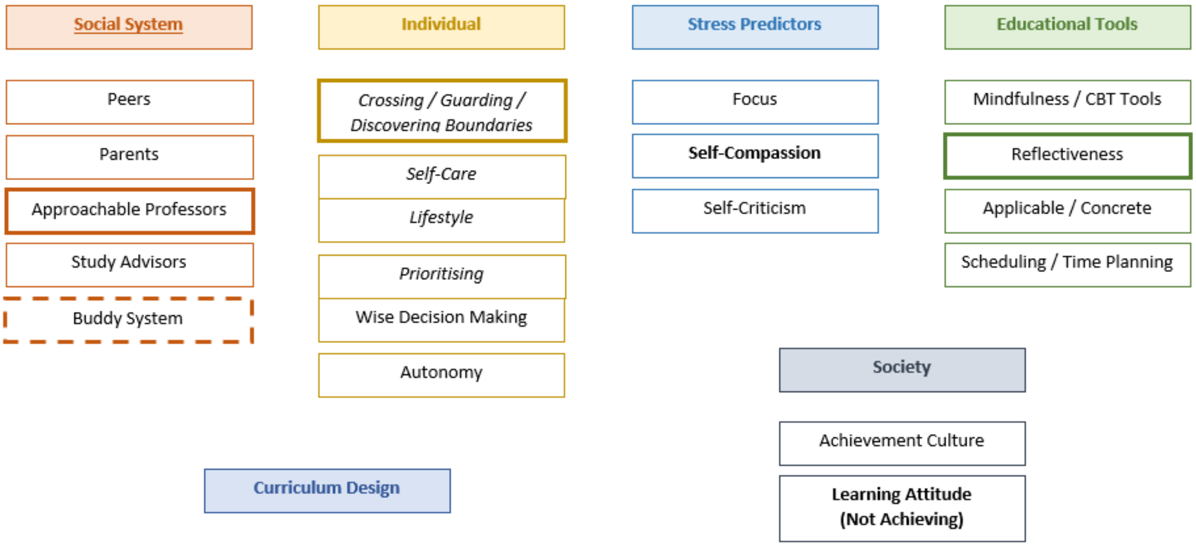
Social System
Peers
Parents
Approachable Professors
Study Advisors
Buddy System
Individual
Crossing / Guarding / Discovering Boundaries
Self-Care
Lifestyle
Prioritising
Wise Decision Making
Autonomy
Stress Predictors
Focus
Self-Compassion
Self-Criticism
Educational Tools
Mindfulness / CBT Tools
Reflectiveness
Applicable / Concrete
Scheduling / Time Planning
Society
Achievement Culture
Learning Attitude (Not Achieving)
Curriculum Design

(e)

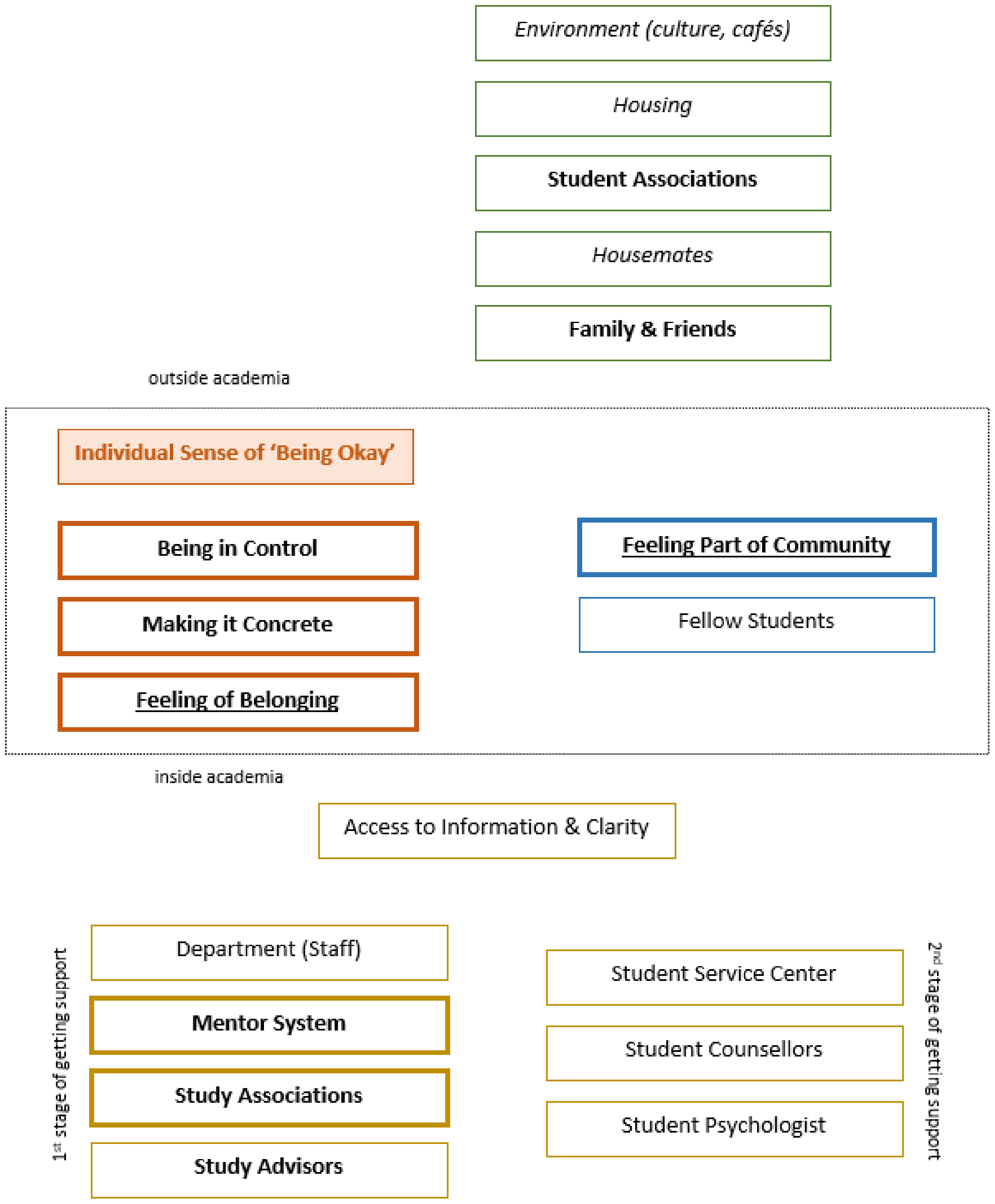
Environment (culture, cafés)
Housing
Student Associations
Housemates
Family & Friends
outside academia
Individual Sense of 'Being Okay'
Being in Control
Making it Concrete
Feeling of Belonging
Feeling Part of Community
Fellow Students
inside academia
Access to Information & Clarity
1st stage of getting support
Department (Staff)
Mentor System
Study Associations
Study Advisors
Student Service Center
Student Counsellors
Student Psychologist
2nd stage of getting support

(f)

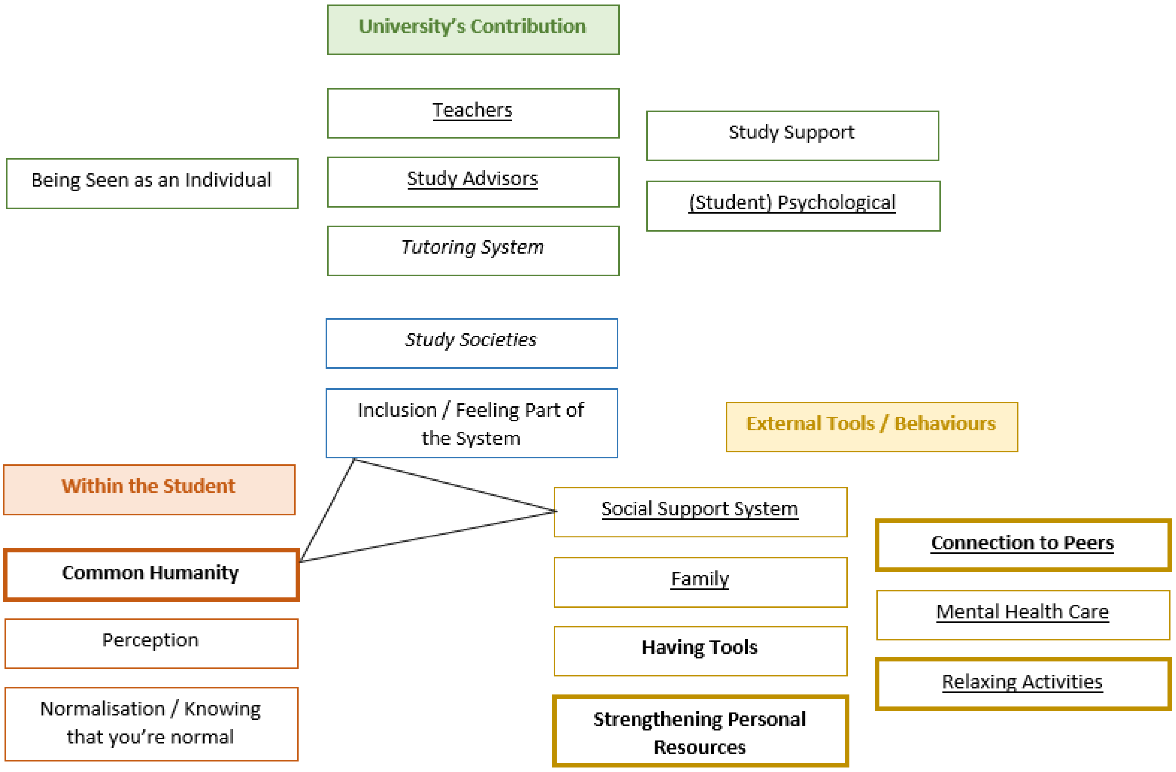

Note. Factors in bold indicate increased importance at t2; factors in italic indicate decreased importance at t2; factors underlined indicate changed in nature at t2; factors with a bold box indicate potential for resilience growth

Figure S2. First comments regarding the preliminary findings of RQ.3 of the students (a, b), a teacher (c), and the study advisor.

(a)

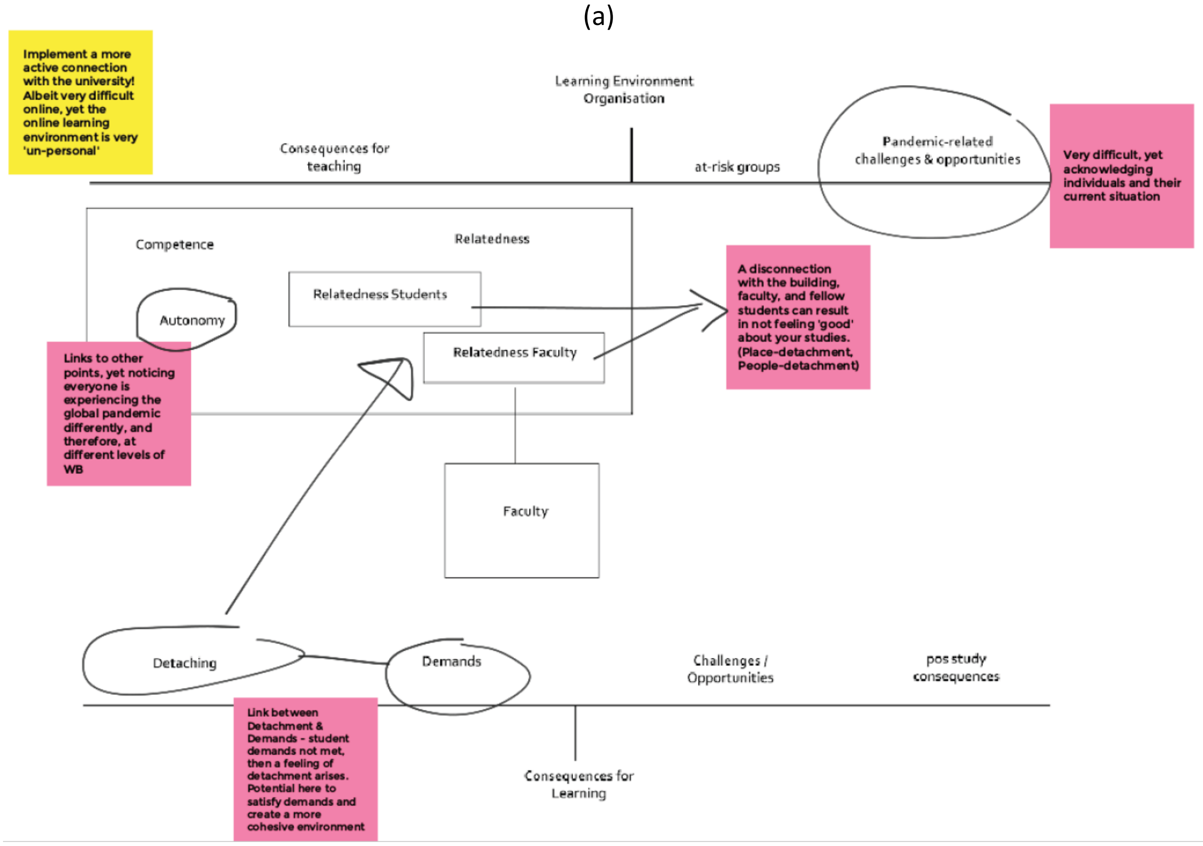
Implement a more active connection with the university! Albeit very difficult online, yet the online learning environment is very 'un-personal'
Learning Environment Organisation
Consequences for teaching
at-risk groups
Pandemic-related challenges & opportunities
Very difficult, yet acknowledging individuals and their current situation
Competence
Relatedness
Relatedness Students
Autonomy
Relatedness Faculty
A disconnection with the building, faculty, and fellow students can result in not feeling 'good' about your studies. (Place-detachment, People-detachment)
Links to other points, yet noticing everyone is experiencing the global pandemic differently, and therefore, at different levels of WB
Faculty
Detaching
Demands
Challenges / Opportunities
pos study consequences
Link between Detachment & Demands - student demands not met, then a feeling of detachment arises. Potential here to satisfy demands and create a more cohesive environment
Consequences for Learning

(b)

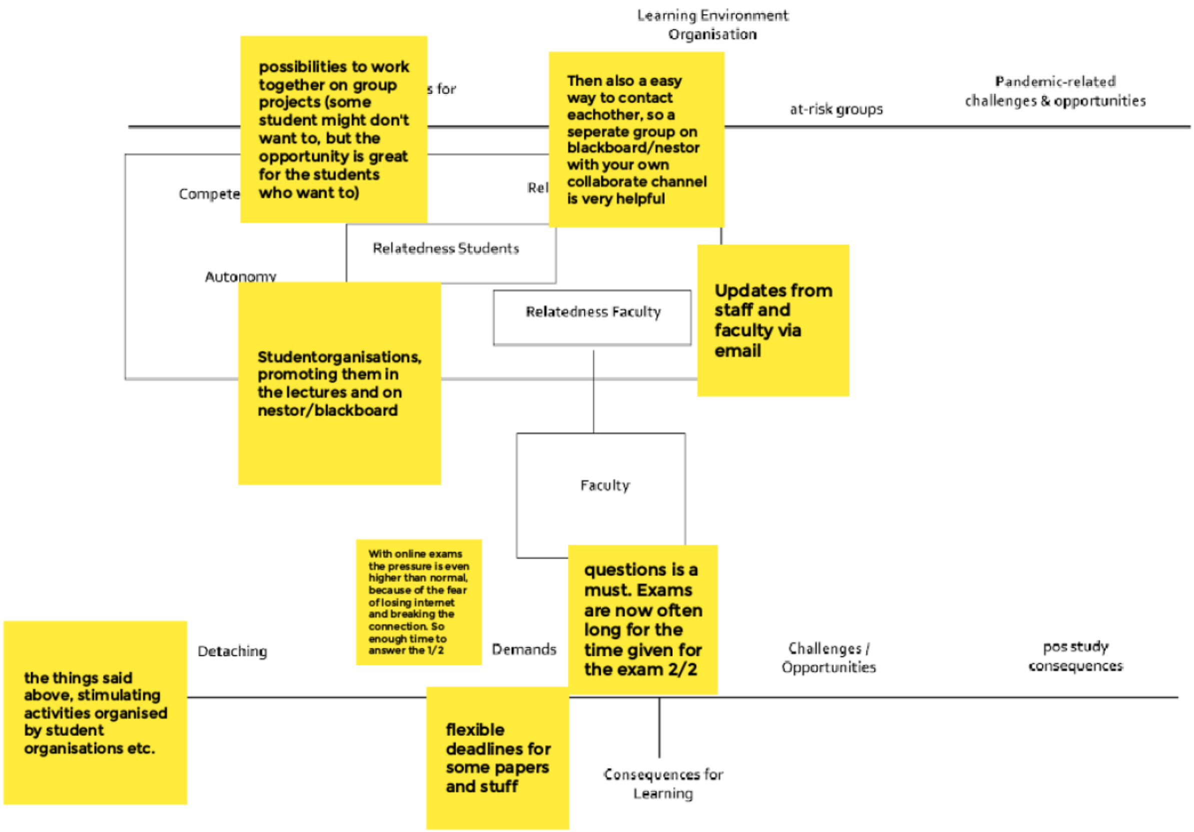
Learning Environment Organisation
possibilities to work together on group projects (some student might don't want to, but the opportunity is great for the students who want to)
s for
Then also a easy way to contact eachother, so a seperate group on blackboard/nestor with your own collaborate channel is very helpful
at-risk groups
Pandemic-related challenges & opportunities
Compete
Rel
Relatedness Students
Autonomy
Relatedness Faculty
Updates from staff and faculty via email
Studentorganisations, promoting them in the lectures and on nestor/blackboard
Faculty
With online exams the pressure is even higher than normal, because of the fear of losing internet and breaking the connection. So enough time to answer the 1/2
questions is a must. Exams are now often long for the time given for the exam 2/2
Detaching
Demands
Challenges / Opportunities
pos study consequences
the things said above, stimulating activities organised by student organisations etc.
flexible deadlines for some papers and stuff
Consequences for Learning

(c)

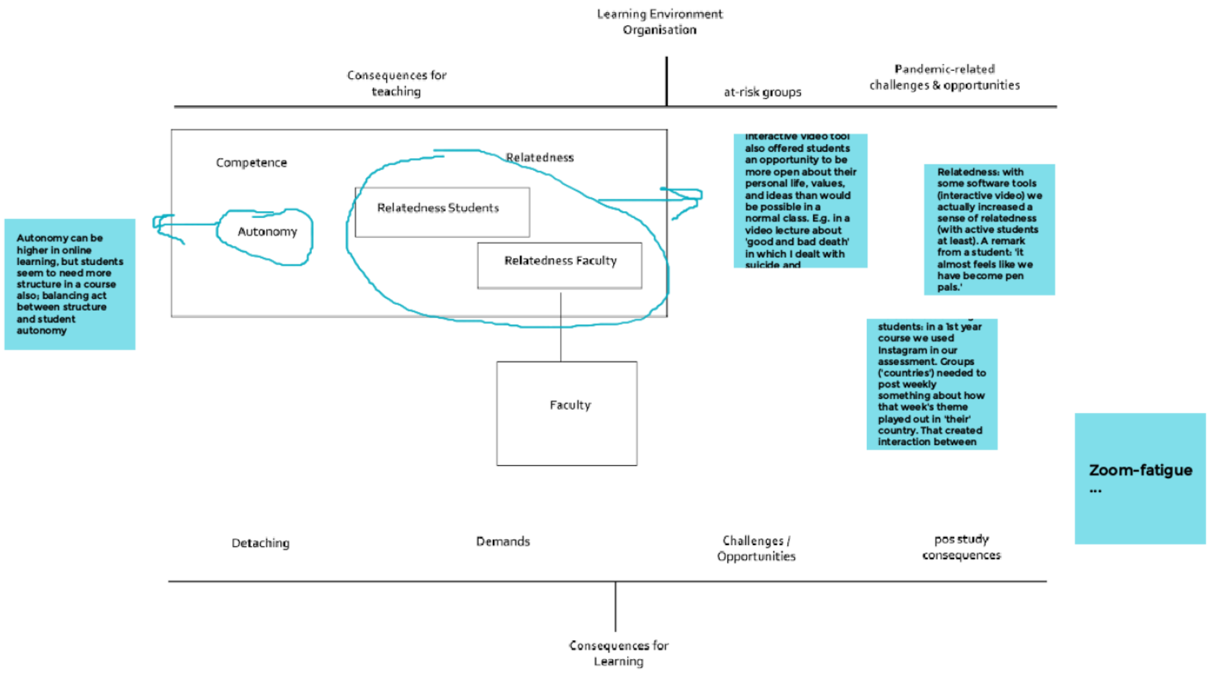
Learning Environment
Organisation
Consequences for
teaching
at-risk groups
Pandemic-related
challenges & opportunities
Competence
Relatedness
Relatedness Students
Autonomy
Relatedness Faculty
Faculty
Autonomy can be higher in online learning, but students seem to need more structure in a course also; balancing act between structure and student autonomy
also offered students an opportunity to be more open about their personal life, values, and ideas than would be possible in a normal class. E.g. in a video lecture about 'good and bad death' in which I dealt with
Relatedness: with some software tools (interactive video) we actually increased a sense of relatedness (with active students at least). A remark from a student: 'it almost feels like we have become pen pals.'
students: in a 1st year course we used Instagram in our assessment. Groups ('countries') needed to post weekly something about how that week's theme played out in 'their' country. That created interaction between
Zoom-fatigue
...
Detaching
Demands
Challenges /
Opportunities
pos study
consequences
Consequences for
Learning

(d)

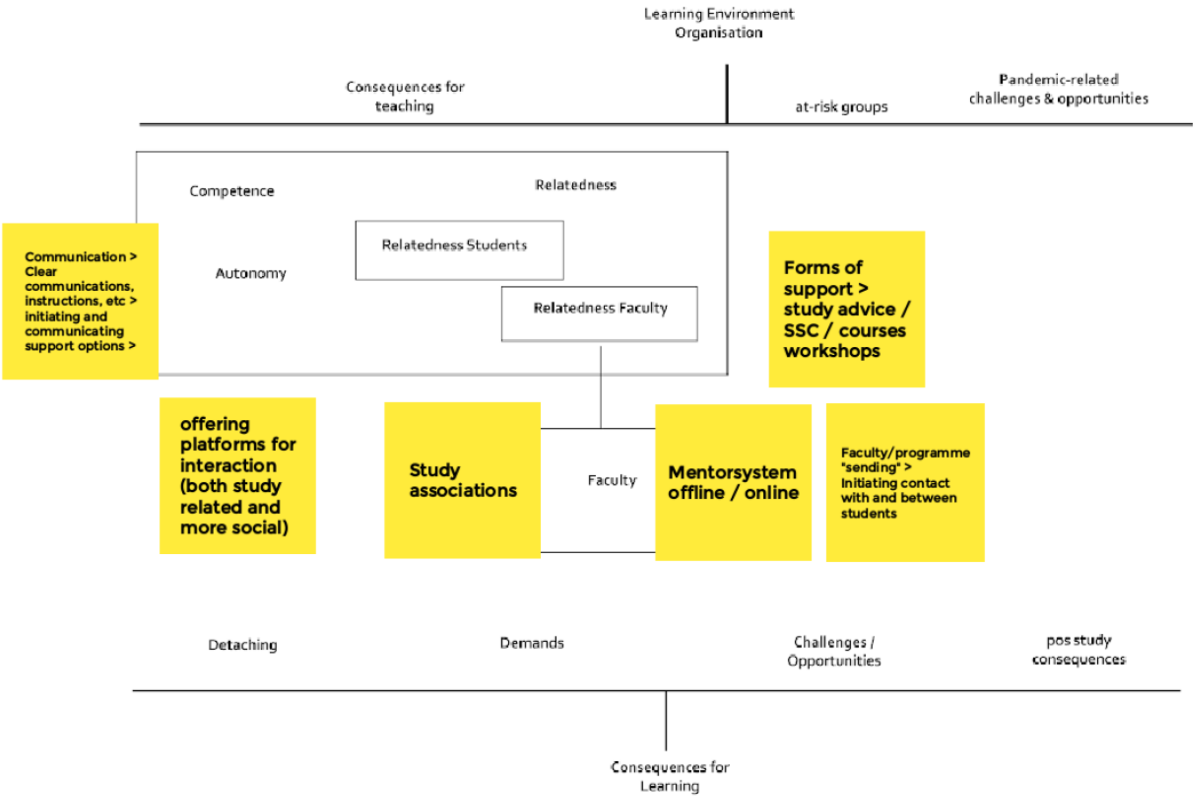
Learning Environment
Organisation
Consequences for
teaching
at-risk groups
Pandemic-related
challenges & opportunities
Competence
Relatedness
Relatedness Students
Autonomy
Relatedness Faculty
Communication > Clear communications, instructions, etc > initiating and communicating support options >
Forms of support > study advice / SSC / courses workshops
offering platforms for interaction (both study related and more social)
Study associations
Faculty
Mentorsystem offline / online
Faculty/programme "sending" > Initiating contact with and between students
Detaching
Demands
Challenges /
Opportunities
pos study
consequences
Consequences for
Learning
