# Supplementary material for: Caught between relief and unease: How university students’ well-being relates to their learning environment during the COVID-19 pandemic in the Netherlands
Source: PLoS One. 2023 Nov 2;18(11):e0292995. doi: 10.1371/journal.pone.0292995 (PMC10621861; doi:10.1371/journal.pone.0292995)
Supplement: S3 File — Including the final codebook (Table S7). (PDF) [file pone.0292995.s003.pdf]

**Supporting Information III – Codebook**

*Final Codebook*

Table S7 displays the complete finale codebook, including explanations and example quotes for each code, alphabetically sorted.

**Tables**

**Table S7.** ***Final codebook including descriptions and example quotes.***

| Codes RQ.1 | Description | Example in data |
|---|---|---|
| ACADEMIC SUPPORT SYSTEM | People within academia, including those at the first line of students' support (faculty, including mentors, teachers, tutors, and further support staff), on the second line of students' support (study support staff, including support groups and study advisors), and on the third line of students' support (mental health support staff, including student psychologists and the Student Service Center). Moreover, this code covers statements on the access to these support systems. | I think my Spanish teacher, she, what's the word, not directly, indirectly influenced my well-being in a negative way. (university student) |
| ADAPTATION | Referring to the process of adaption to the new situation and environment, including personality traits such as being adaptive next to the process of adaption comprising adjustment stress, becoming used to it, and finally acceptance | Um, and then once I accepted that it wasn't gonna happen, then it was okay. Um, because I once still really wanted that (university student) |
| ADAPTION AT UNIVERSITY LEVEL | Referring to the process of adaption to the new situation at a university level in terms of the teachers adjusting to the pandemic-related surroundings compared with the usual academic surroundings, including adjustment also on a curriculum level and the institution's (re-)organisation | and I'm-think the uni adapted very quickly and really well. So, I feel positive about that (university student) |
| AT-RISK GROUPS | Referring to potential student subgroups especially at risk with emergency remote teaching, for instance first year or international students due to, amongst others, time differences | particular for the newly starting ones, the first years. Yah. [...] fun. Well, it's worse-worse for them. It-it's the same for-for all the others of course, but, it's worse, if you haven't met anyone and you don't get the chance to meet them. And, uh, once a week, two hours, meeting or whatever, that-that's not being a student, so. (university teacher) |
| AUTONOMY | Referring to students' experience or lack of autonomy and the learning environments' potential to satisfy students' need of autonomy, such as when flexibility | Um, well (p) I guess, um, at least in-on the flexibility side, yah, a lot of students have side jobs and what not - when lectures are being offered online, they're not from 10 to 12 necessarily, but for |

| | | |
|---|---|---|
| | and transparency gives them a feeling of being in control. | example they're, you can [inaudible], they're open online for a day or a week or even forever. Um, it will become more easy to, um, well, again, manage your week, plan your week. Um, which will, I guess, give an added sense of being in control (study advisor) |
| CHALLENGES & OPPORTUNITIES | Referring to challenges and opportunities on several levels. First, on a global or institutional level, such as creating a new normal with new possibilities and having understood how crisis within education works. Secondly, the code refers to colleges and opportunities within the classroom on an individual level, depending on if one grasps the opportunity to grow in corresponding aspects, such as own flexibility and responsibility or self-structuredness and – discipline also including difficulties surrounding conveying non-knowledge related skills. The latter needs to be distinguished from outcomes, as these became more and more relevant and were thus seen as a single code closely related to outcomes. | Um, being more able to, uh, manage their own lives and the times, getting structure in, well, in your days basically and make enough time for studying. (study advisor) |
| COMPETENCE | Referring to students' lack of sense of competence and the learning environments' potential to satisfy students' need of sense of competence, by giving them structure to give them a feeling of productivity. | And the same with self-competence, especially when it comes to studying, I think that's lacking for a lot of students and that's what they struggle with a lot. (student psychologist) |
| CONTEXTUAL FACTORS | Contextual aspects that influence students' well-being, such as the mental health care outside of the academic world, the achievement culture, as well as students outer environment, including housing. | I: Or anything that- F: -strikes me as missing. (pauses) Well, maybe just the mental health care just as a- (p) (student psychologist) |
| DAILY ROUTINES | Referring to the struggles with losing one's daily structure and routines, including the feeling of not being able to continue one's daily life and experiencing the days as a 'blur', but also the process of creating structure and daily routines to cope with these struggles | And then I, well, the thesis was going and stuff, so I had to work at my thesis and you, uh, I think I got a bit used to the routine and stuff, just with the housemates, we-usually we are working for ourselves and then at the evenings we eat together and are watching TV or something together and then that became, yah, that became more of a |

| | | |
|---|---|---|
| | | routine. That helped a bit, I think, uh, and-yeah<br>(university student) |
| DEMANDS | Referring to the demands that are made for the students, such as keeping the pace & quality of education. Additionally, the code refers to the consequences thereof, such as concentration problems, and heightened workload as well as consequences of if these demands were not made, including study delays. | the students feel a bit more pressure. And I told-I experience myself as well, and I-I talk with my fellow students a lot, they say: 'Well, the university is-is expecting, well, that we-we got a few weeks extra, but, they're expecting that we're just as productive as we're working at home as we're working when the situation was normal'. And, especially for me it doesn't work that, I think, uh, for the other students it's, uh, it doesn't as well, so, well.<br>(university student) |
| DETACHING | Referring to the process of detaching from one's student role, including the feeling of 'closing a chapter' and the lack of purpose when studying. The code also refers to students' engagement, motivation, and participation in classroom. The code needs to be distinguished from outcomes, as it became more and more relevant and was thus seen as a single code closely related to outcomes. | I think, when-so, they kind of got torn out of their student bubble, I'd say, yah, yah, and dropped in reality, and in the Netherlands you can easily stay in a bubble for a long time<br>(university teacher) |
| DISRUPTION OF SOCIAL LIFE | Referring to the effects from social distancing. These include how the social contact has changed, as well as comments on social pressure and social well-being in general. More specifically, the code also covers the experiences of international students and how these had to adjust to the disruption to their social lives, including the potential realization that they have missed their original home all along | K: […] but I think there are subgroups that have it more difficult. So,-<br>I: Which ones? For example?<br>K: Yah, yah, good question. I think the ones that really need other people<br>(university teacher) |
| EDUCATIONAL TOOLS | Tools that, for instances, teacher use to enhance well-being, including curriculum design and examination methods, including tools that fail to enhance well-being. In this, educational tools are distinguishable to the learning environment organisation as they depict specific tools instead of a general set-up | I've quite a-in most of my courses I have different types of assessment. And use rubrics these days to do the assessment on whatever. […] Sometimes students get stressed out because they get a-get a fail on one of these partial grades. And they can always compensate.<br>(university, teacher) |
| FACULTY | Referring to the role of the faculty in this regard, their well-being and | Um, if all my courses will be, um, indicated as being ones that can |

| | | |
|---|---|---|
| | adaptation struggles, the support they need, and their (lack of) development | be taught online, it's not fun, that's what I wouldn't like, but yah (university teacher) |
| FINANCIAL INSECURITY | Referring to students having trouble with keeping up their financial obligations while losing their jobs due to the pandemic | And they, oh, sometimes international students come with funding, and then the funding stops, so, I think stressors like that. (university teacher) |
| GENERAL LEARNING STRATEGIES | Strategies the students have developed and used to enhance their studying and, hence, also their well-being, such as concentration strategies and prioritising. | So those who are better capable of focusing and are more goal-directed experience less distress. (university teacher) |
| INDIVIDUAL RESOURCES | Resources the individuals have developed or can rely on to remain a certain level of well-being or regain it after adversities. This includes hobbies such as sports, recreation, and relaxation, but also personal resources such as self-care/-compassion and reflectiveness, leading to the process of crossing, guarding and discovering boundaries and knowing oneself. Moreover, this code refers to the students' study attitude, wise decision making, and beliefs, including a feeling of normalization of negative well-being, the realization that their study outcomes do not define who they are and a sense of common humanity. | yeah, my first, um, question would be um, to make the-to have the student make it a bit more concrete, what are you distressed about? Yeah. Because that you need to know in order to do something and-or take action on improving the situation. (study advisor) |
| LEARNING ENVIRONMENT ORGANISATION | Referring to the various potential learning environment set ups, ranging from online to hybrid to offline environments. The code also refers to the aspects that shape the new learning environment, including the COVID measures on a university level one has to adhere to, the adjusted course size, the university's transparent communication and aspects surrounding home office. In this, the learning environment organisation is distinguishable to educational tools as it depicts a general set-up instead of specific tools. | Um, uh, so that was nice. Um, they - the introduction week they kind of tried to do that, but it didn't really work, because, I mean, no one really knew each other, and you're all behind a screen, um, so they haven't really done anything, um, which is a shame. (university student) |
| LOSS OF CONTROL | Referring to the general sense of loss of control, but also the attempts to regaining control | because I think, autonomy here, especially for students, I think, there's a lot that they can't |

| | | |
|---|---|---|
| | | control right now, so that hacks into their feelings of, uh, autonomy, I think. Um, there's a lot out of their control. (student psychologist |
| LOSS OF DEVELOPMENT | Referring to the general sense of loss of development, of being in a standstill and not developing academically but also personally | You have three or four years of your life in a period in which you develop yourself quickly. And it-it's more or less wasted these months. They've learned something, they've got their ECs or whatever, but, it's-it's not the self-development that you want, the interaction with other people. And I-that-that can't be good for your well-being, I would say, but, I don't know. (university teacher) |
| LOSS OF EXPERIENCE | Referring to the general sense of loss of excitement and experiences that students now miss out of | and it was still difficult, like, knowing I'd miss out on the, like, last four months of summer, here, as a student and finishing off my degree here and stuff, um, so that was sad. (university student) |
| MINDFULNESS | Referring to the positive aspects of the pandemic in terms of slowing down, regaining one's balance, and realizing that one can be happy with what one has | Uh, the benefits are probably they had to juggle less in their life. So, um, and what-what I saw before is that students had so money-many obligations, uh, not only study-related, but also student life-related, friends-related, work, and all that. It might be a bit-the pace has gone down a bit. Not the edge of the stress levels. And that's-that helps students to keep a better balance. (university teacher) |
| OUTCOMES | Referring to the process of perceiving one's own progress, as well as one's engagement, commitment, academic satisfaction | We started with progress testing in our Bachelor program which is forwarding [if?] testings and one of the purposes I have in mind with it is (p) it's an idea with me and one of my colleagues what we want is students, show them that they are really making progress. Because you are not aware of it if you are in the system, but- (study advisor) |
| PANDEMIC | Referring to everything related to the pandemic, including measurement adherence, crisis-related struggles, quarantine and isolation, as well as coronavirusanxiety. Moreover, the code covers the role the | Yah, but, if you-if you're really scared of the virus, maybe, because you have parents with-with underlying conditions or you have this for yourself, and I think it's-it's more of a step to find a new job or something, but, |

| | | |
|---|---|---|
| | pandemic plays in counselling sessions. | because at most jobs you are at higher risk because you're close to people and stuff. Yah, it's difficult, I think, I don't know (university student) |
| POSITIVE STUDY CONSEQUENCES | Referring to positive outcomes from emergency remote teaching for the individual, such as finding joy in studying and having more times to study. The code needs to be distinguished from outcomes, as it became more and more relevant and was thus seen as a single code closely related to outcomes. | also from what I hear from certain students, um, might benefit from on that point. Um, I've been talking to students who say: 'Well, I haven't been so concentrated and focused on my studies, I never had so much time to actually, well, to work on the stuff I have to do for my online classes.' (study advisor) |
| RELATEDNESS FACULTY | Referring to the interaction and relationship between students and faculty, especially teachers, given emergency remote teaching, including the observation of increasing distance between the two parties. Furthermore, the code refers to everything that defines the student-teacher relationship in that way that it may or may not enhance student well-being. That includes acknowledgement and appreciation of each other(‘s struggles), potential mistrust (regarding online exams), giving them a feeling of autonomy and being as concrete and applicable as possible, but also establishing an open relationship, seeing them as an individual, and providing them with the feeling that they’re doing good. | because I don't know anything, don't hear anything [...] the last, I- the last time we spoke I was in the middle of teaching three courses of […] had more contact with students, and now with this one course and five students present only, but - I don't know. I simply don't know, and that's the problem (chuckles). We're guessing about each other's well-being, yah (university teacher) |
| RELATEDNESS STUDENTS | Referring to the interaction and relationship amongst students, given emergency remote teaching, including the interpersonal climate | K: I-I think it's, for some students it's very hard, I think. Uh-<br>I: Why?<br>K: Ah, well, because their-all of the online education, not integrating among each other (university teacher) |
| RESILIENCE | Referring to concrete statements about students’ or faculty’s resilience, hence, their strength and personal resources that help them keep psychologically healthy despite the pandemic. | I think for the long-term, so, I gave this a lot of thought actually and I didn't share too much with others, it's been more in my own thinking - I think for the long-term, for the development for people, having experienced this, is very healthy, actually. (university teacher) |
| RESILIENCE GROWTH | Referring to statements on resilience growth, hence, a feeling of added strength and personal | […] one thing that made me cope better with my well-being was having like, like a few really shit, |

| | | |
|---|---|---|
| | resources because of having experienced adversity. | awful things happen, and then like, and then getting mentally stronger from that.<br>(university student) |
| SENSE OF BELONGING | Referring to feeling part of a community and the sense of belonging to one's study environment, including the buildings, institution, faculty, programme, and fellow students. | I think feeling part of a system is important.<br>(student psychologist) |
| SOCIAL RESOURCES | People who provide students with a sense of social support and connection, including their friends, fellow students, student associations, and family, but also processes related to this, such as socializing. | I-eh-we as former board members, we've become a very strong group, we, um, um, we will eat together every week and, eh, well, they're still there if-if sometime-if there is um, there is sometimes, um, you are struggling with something and they know you so well […]<br>(university student) |
| SOCIETY | Referring to the role the society plays in the students' experiences of the pandemic | I think what would be good and on a really grand scale, and that's more environmental, our movement as a society, the flying, the travelling, and things like that. It would be really good for the environment to keep that.<br>(university teacher) |
| UNCERTAINTY | Referring to the uncertainty surrounding a pandemic, including the abruptness with which the pandemic hit, but also the confidence one has concerning potential improvements and the assumptions about the future. | I think they were very early with cancelling EVERYTHING until first of September, I think that was too early. But on the other hand, it did give, um, staff and students, um, clarity, if that is the correct word. It was immediately clear: 'Well, we switch to online, we keep on doing that', so<br>(university teacher) |
| WELL-BEING | Referring to students' well-being, including the context that determines their well-being and if their well-being has been stable or amplified. Moreover, the code refers to awareness raising of the importance of well-being, and physical health, but also students' need and search for as well as access to mental health services. | I'm inclined to s-but I don't know any numbers, but I'm inclined to say that it has, um, it's gone back to normal in that aspect as well a little bit, because, for instance with social anxiety, things are starting up again, with burn out symptoms, pressures rising again, so I think that's coming back now, but, overall, no, it hasn't changed that much I think.<br>(student psychologist) |
| WELL-BEING NEGATIVE | Referring to statements on the negative development of students' well-being. Furthermore, students' lack of energy is also coded within this category. Finally, the code covers students negative emotions. | Um, again, for another group of students it might be a bit of a black hole, um, where they get sucked into, with isolation of, uh, not doing anything, um, yah, of not getting, um, grip on the situation and-and making it work |

| | | |
|---|---|---|
| | | the best as possible, considering the circumstances for themselves. (study advisor) |
| WELL-BEING POSITIVE | Referring to statements on the positive development of students' well-being. Furthermore, students' energy is also coded within this category. Finally, the code covers students positive emotions. | Yah, I hope so, yah. And I generally think, um, that there probably is a slight increase in student well-being over the last couple of, well, over the Corona period, yah. (study advisor) |
