# Supplementary material for: Caught between relief and unease: How university students’ well-being relates to their learning environment during the COVID-19 pandemic in the Netherlands
Source: PLoS One. 2023 Nov 2;18(11):e0292995. doi: 10.1371/journal.pone.0292995 (PMC10621861; doi:10.1371/journal.pone.0292995)
Supplement: Attachment — Submitted filename: Response to Reviewers.docx [file pone.0292995.s004.docx]

Reviewer #1:

**Thank you for giving me the chance to review the manuscript "Caught between relief and unease: How university students’ well-being relates to their learning environment during the COVID-19 pandemic in the Netherlands:. The authors did a great job in their analysis and writing. I have very minor comments:**

**Introduction:**

**I don’t necessarily agree with line 79. There have been ample studies published on resilience during COVID-19, that target different populations or groups of individuals. To mention few:**

**Barzilay, R., Moore, T.M., Greenberg, D.M. et al. Resilience, COVID-19-related stress, anxiety and depression during the pandemic in a large population enriched for healthcare providers. Transl Psychiatry 10, 291 (2020). https://doi.org/10.1038/s41398-020-00982-4**

**Labrague, LJ, de los Santos, JA. COVID-19 anxiety among front-line nurses: Predictive role of organisational support, personal resilience and social support. J Nurs Manag. 2020; 28: 1653– 1661. https://doi.org/10.1111/jonm.13121**

**Ye, Z., Yang, X., Zeng, C., Wang, Y., Shen, Z., Li, X. and Lin, D. (2020), Resilience, Social Support, and Coping as Mediators between COVID-19-related Stressful Experiences and Acute Stress Disorder among College Students in China. Appl Psychol Health Well-Being, 12: 1074-1094. https://doi.org/10.1111/aphw.12211**

**Haldane, V., De Foo, C., Abdalla, S.M. et al. Health systems resilience in managing the COVID-19 pandemic: lessons from 28 countries. Nat Med 27, 964–980 (2021). https://doi.org/10.1038/s41591-021-01381-y**

**Prime, H., Wade, M., & Browne, D. T. (2020). Risk and resilience in family well-being during the COVID-19 pandemic. American Psychologist, 75(5), 631–643. https://doi.org/10.1037/amp0000660**

Dear reviewer, first of all thank you for taking the time to review our manuscript and being positive about it!

Thanks for pointing us to more literature about resilience during COVID-19. We agree that the sentence no longer holds and deleted it instead. Additionally, we decided to add the study by Barzilay et al. (2020) as we considered it suitable for our study’s context; the study by Ye et al. (2020) was indeed already cited in the paragraph.

*For instance, more resilient students perceive less stress during COVID-19.[7] Likewise, students’ resilience moderates pandemic-related stressful experiences and acute stress disorder.[26] Also in the general population, more resilient individuals experienced fewer worries, anxiety, or depression symptoms. [27]*

**Discussion:**

**The discussion would benefit from mentioning the findings of:**

**Ang, W., Shorey, S., Lopez, V. et al. Generation Z undergraduate students’ resilience during the COVID-19 pandemic: a qualitative study. Curr Psychol (2021). https://doi.org/10.1007/s12144-021-01830-4**

**Prieto, D., Tricio, J., Cáceres, F., Param, F., Meléndez, C., Vásquez, P. and Prada, P. (2021), Academics' and students' experiences in a chilean dental school during the COVID-19 pandemic: A qualitative study. Eur J Dent Educ, 25: 689-697.** [**https://doi.org/10.1111/eje.12647**](https://doi.org/10.1111/eje.12647)

Thank you for your nice suggestion. We read the two papers and agree that they are very fitting and relevant for our study and incorporated them in our discussion (see revised manuscript).

**Other comments:**

**Line 69: may “Counteract” - please ensure all text is checked for spelling and grammar**

Thank you for making us aware of this mistake. We corrected it and additionally checked the whole document once again using a spelling and grammar tool and hope that it is now without any language errors.

**Figure 1: what does UG stand for?**

UG stands for the University of Groningen, which we anonymized for the first round of revisions. We now added a respective explanation to the note of the figure, so that it should be clearer now.

Again thank you very much for your help regarding our manuscript!

Reviewer #2:

**In this qualitative study the authors attempted to assess whether the COVID-19 pandemic affected on students’ well-being, relevant resilience factors and the role of the learning environment in this regard.**

**They enrolled six participants who were administered a semi-structured guide at four different time, founding evidence of the impact of the pandemic on all the three aspects investigated. Hence, the authors suggest a reshape of the academic system according to their findings.**

**The article is interesting and may provide useful information, but I suggest some revisions:**

**1-I suggest revising the methodology section. It would be useful to specify more in details the time period in which participants were recruited and the timing with which the semi-structured interview was administered (not only in Figure 1). Likewise, it would be important to make a summary of the tables found in the supporting information in the methodology paragraph, to better understand what it was asked to the participants in the different timeframes.**

Dear reviewer; first of all thank you for your generally positive feedback and the time you took to give suggestions to improve the manuscript.

We agree that more specific information about the time period would be useful to understand the context the study is set in, which is why we included Figure 1 in the first place. We have now elaborated on this and also added some information in written form:

*As the situation changed quickly during these months, it appears essential to understand the situational context (see Figure 1). We recruited the participants at the end of 2019 and interviewed them for the first time before the pandemic hit the Netherlands. The second time of data collection then fell in the period of the first lockdown and the end of the academic year, with social distancing measures and the closure of universities in place. The third interviews, however, took place in the summer holidays, during which normality had somewhat returned, although remaining socially restricted. Lastly, t4 was similarly affected as t2 by the second lockdown in the Netherlands whilst the new academic year had just begun.*

Beyond that, we agree that additional information concerning the interview schedules improves the manuscript and added a respective summarised table to the main text as well.

**2-In my opinion, it is necessary to explain in more detail the limitations of the study. First, it is important to point out what are the weaknesses of a qualitative study. Secondly, I suggest that more emphasis should be placed on the smallness of the sample and all that goes with it.**

Thank you for the remark. We agree that more emphasis on the limitations helps understand the interpretability of the study better. This is why we expanded this section:

*Although the findings contribute to a deeper understanding of the interaction between students’ well-being and the LE, certain limitations remain. These mainly regard the rather small sample. Interviewing only two students may have excluded essential aspects, such as a precarious financial situation. Also in general, a sample size of only six participants has most likely restricted the representability of this study, as it encompasses too few individual experiences. A sample size of 15 to 20 interviewees would be preferable for qualitative research, though prior research also indicates that 12 participants can suffice and achieve saturation when using thematic coding, and as few as 6 participants may already cover essential elements.[65] For our study, the six interviewees participated four times throughout the study, adding up to 24 interviews in total. Consequently, the present findings may be grounded in sufficient data to ensure basic saturation regardless. Moreover, the qualitative nature of the study in itself may have been an additional limitation. Whereas the detailedness of individual experiences belongs to the strengths of qualitative research, it automatically comes with a lack of generalisability to a greater population.*

**3-Lines 444-444: “Therefore, universities must attempt to balance the positive and negative consequences that online eaching entails”. It would be helpful to explain in further detail the positive and negative aspects of online education with a more comprehensive survey of the available literature.**

Thank you for the remark and we agree that the paragraph benefits from more detail. This is why we added additional literature to the paragraph to elaborate on both the positive and negative consequences of online teaching.

*Ultimately, we found both negative and positive consequences for online learning, revealing the complex aftermath of the pandemic that academia must take into account. Despite positive aspects, previous research has demonstrated that students generally prefer offline to online education,[52] and that studying online is unfavourable for their resilience development.[64] Other researchers investigating academic well-being throughout COVID-19 have found this duality as well.[Ferrer,Prieto] According to these findings, advantages encompassed shorter travel time, easier access to education and increased student involvementthe possibility to study according to one’s personal learning style., whereasAt the same time, disadvantages spanned depersonalisation, screen fatigue, and technical difficulties.[Eringfeld,Ferrer,Prieto] Therefore, universities must attempt to balance the positive and negative consequences that online teaching entails.*

Thanks again for your time and effort to help us improve this manuscript!
