# Supplementary material for: Caught between relief and unease: How university students’ well-being relates to their learning environment during the COVID-19 pandemic in the Netherlands
Source: PLoS One. 2023 Nov 2;18(11):e0292995. doi: 10.1371/journal.pone.0292995 (PMC10621861; doi:10.1371/journal.pone.0292995)
Supplement: Attachment — Submitted filename: Response to Reviewers.docx [file pone.0292995.s005.docx]

Reviewer #3:

**The manuscript "Caught between relief and unease: How university students’ well-being relates to their learning environment during the COVID-19 pandemic in the Netherlands” has an interesting topic and can bring new insights about factors contributing to university students’ well-being and the development of students’ resilience during the pandemic. While the overall writing is excellent, there are some issues that need to be addressed.**

**First, because of the very small sample size the study should more strongly focus on the longitudinal data. Especially a comparison of pre-COVID-data with data collected during the pandemic might be very informative.**

Dear reviewer, first of all thank you for taking the time to review our manuscript and being positive about it!

We agree that the longitudinal data constitutes a strong point of our study and emphasized the findings surrounding the developments throughout the pandemic. Therefore, we added additional longitudinal information that we omitted previously due to the word count. For instance:

*Moreover, participants reported trends in student well-being, such as whether it had amplified, stayed stable, decreased, or increased. A university teacher stated at t3 that she was “positively surprised” to see her students coping so well, for example. Whereas participants remained neutral when talking about well-being before the pandemic hit, they got more specific throughout the pandemic, frequently differentiating between negative and positive well-being. Generally though, negative statements about student well-being outweighed the positive ones across all time points, stated mainly by the students.*

and:

*Considering the longitudinal development of the experiences of these stressors, participants reported disruption of social life, uncertainty, and daily routines throughout the whole pandemic period, yet with particular emphasis shortly after the pandemic hit (t2). At the same time, they reported having become used to and having accepted the situation at t3 and t4, respectively, which might explain the decreasing emphasis on the pandemic-related stressors.*

**Second, the presentation of the results could be optimized:**

**Table 2: I was irritated because the text uses a different wording from the table. Are personal resources (as mentioned in the text) and individual resources equivalent?**

We apologise for the ambiguities. The table should indeed display ‘personal resources’ instead of ‘individual resources’ – we adapted the table accordingly.

**In this same vein: I am missing a clear description of the outcome-categories/sub-factors as well as a delimitation of the categories from each other. There seems to be some overlap between the categories/sub-factors. This makes the results hard to understand. There is a somewhat clearer description in the discussion section, but an exact description of the categories/sub-factors needs to be provided in the results-section.**

We agree that the results are complicated to disentangle, particularly due to the overlap between categories noted by the reviewer. We now rewrote the section for more clarity and added sub headings to structure it better. For instance:

*For student well-being, we identified well-being itself as a cluster, both in a positive and negative sense as two subfactors. This cluster comprises heightened awareness of mental health on the positive side, as well as students’ physical health, fatigue, and delays in seeking help on the negative side.*

Additionally, we added a clarification paragraph at the beginning of the results section:

*In the following, we will give an overview over the clusters we identified throughout the analysis, structured according to the three research questions. We differentiate between clusters – an overall theme of various codes – and subfactors – more detailed themes we found within these clusters. Figure 2 depicts an overview of these clusters (displayed in bold) and potential subfactors (displayed in regular font).*

**I am also missing information concerning the development of resilience that go beyond the participants explicit statements but may be evident from the longitudinal data.**

We tried to incorporate a more elaborate comment on the longitudinal development of resilience beyond the participants’ statement:

*Third, interviewees highlighted reinforced resilience resources due to prior adversity, which led to a strengthened self in the present, and lastly, they commented on their potential to grow their resilience by experiencing the current crisis. This differentiation between resilience due to prior adversity and resilience growth due to the pandemic itself also echoed the longitudinal data: Whereas participants mentioned resilience as a relevant well-being resource already before the pandemic, they only mention the process of resilience growth itself as the pandemic progressed, particularly during the second infection wave (t4).*

We hope that our adjustments in general and specifically for the development of resilience clarify and emphasise the longitudinal results better!

**Minor issues:**

**p.1, 48-49: “For instance, governments introduced persisting restrictions on social and public life that have substantially reshaped the academic learning environment (LE), defined as stakeholders, and educational and structural aspects” Is “[…] defined as stakeholders, and educational and structural aspects” the definition of an academic learning environment? If so, this is not readily intelligible. Perhaps you can find a wording that is more easily understandable.**

We adjusted the passage and hope that it is now clearer what exactly we meant by it:

*For instance, governments introduced persisting restrictions on social and public life that have substantially reshaped the academic learning environment (LE). For the scope of the current study, we define the LE as an environment in which stakeholders act (e.g., students, teachers, and support staff), and in which educational (e.g., teaching modes) and structural aspects (e.g., support systems) play crucial roles. As university students, hence, faced unexpected remote teaching, social distancing, and lockdown within their LE, their well-being and resilience resources may have been impacted.*

**p.2, 77-78 I believe this sentence should be in the past tense, as is the following sentence.**

That is true, thank you for pointing it out. We adjusted the sentences accordingly.

**p.6 128- 130 “Many students thus have experienced decreased motivation and engagement (lack of autonomy), unproductiveness, mental overload (lack of competence), as well as isolation (lack of relatedness).” The relation of the bracket expressions to the text is not obvious. I think that the authors mean that decreases in motivation is due to a lack of autonomy support and so on. Please specify, to make this obvious for the reader.**

We adjusted the passage to clarify that these aspects lead to a lack of need satisfaction:

*These aspects may add up to a lack of need satisfaction: Many students have experienced decreased motivation and engagement (corresponding to a lack of autonomy), unproductiveness, mental overload (corresponding to a lack of competence), as well as isolation (corresponding to a lack of relatedness).[18,52]*

**p.6 130-131 “Students’ relationships with peers and teachers have especially suffered.[53,54]” You might add a quantitative study here, e.g.**

**Müller, F.H., Thomas, A.E., Carmignola, M., Dittrich, A.-K., Eckes, A., Großmann, N., Martinek, D., Wilde, M. & Bieg, S. (2021) University Students’ Basic Psychological Needs, Motivation, and Vitality Before and During COVID-19: A Self-Determination Theory Approach. Frontiers in Psychology, 12:775804.**

Thank you for pointing out the study by Müller et al; it is very fitting indeed! We added it to the manuscript and the reference list.

**p.20 410-411 “students mainly mentioned social resources, but university staff considered relatedness with faculty members as more relevant.“ I wonder why relatedness with faculty members is not considered a social resource but considered an academic resilience factor.**

We understand the confusion and tried to clarify the passage to make the differentiation between the two codes clearer, while acknowledging that there is some overlap between them:

*Yet they varied in their perceptions of which other resilience factors were most important: students mainly mentioned social resources concerning relationships outside the academic realm, but university staff considered relatedness with faculty members within academia as more relevant. At this point, we want to acknowledge that both codes overlap, as social resources outside academia may be fellow students that have become friends students meet up with outside university as well. However, we still consider it important to distinguish between the two aspects, as they developed differently throughout the pandemic. For instance, faculty members believed social resources outside academia to be increasingly relevant, whilst relatedness with faculty members lost significance.*

**p.21 439-441 “Although a compromised sense of relatedness and connection should be expected, given social restriction measures, the findings pertaining to growing distances indicate that relatedness has become increasingly disrupted.” This sentence is illogical – starting with ‘although’ I would expect a different outcome instead of findings that corroborate the expectations.**

That is true, thanks for pointing it out! We adjusted the sentence accordingly:

*Given the social restriction measures, a compromised sense of relatedness and connection should be expected; the findings pertaining to growing distances confirm this hypothesis and indicate that relatedness has become increasingly disrupted.*

**p.22 475 “For our study, the six interviewees participated four times throughout the study, adding up to 24 interviews in total.” contradicts p.11 224-226 “To ensure interrater reliability, two independent researchers executed this coding stage, one for the whole data set and the other for 8 of the 20 interviews.”**

Thanks for pointing out this mistake! It should indeed be 24 interviews at p.11, 24-226. We adjusted the text accordingly.

**p.23 “As most studies in this vein have been mainly qualitative,[46] researchers should pursue more quantitative insights, to generalize the results.” I cannot agree with this statement as there are numerous quantitative studies on this topic. You can find many of these papers on** [**https://selfdeterminationtheory.org/topics/application-covid19/**](https://selfdeterminationtheory.org/topics/application-covid19/)

Again, thanks for pointing out additional quantitative studies regarding the topic. We omitted the sentence.
